# Ovarian Dysmenorrhœa

**Published:** 1902-05-24

**Authors:** 


### OVARIAN DYSMENORRHŒA.

An ill-chosen name leads to much mischief, and in medicine may perpetuate much false pathology. As bearing upon this we would draw attention to some remarks recently made by Dr. Herman[1] on the subject of "ovarian dysmenorrhœa." In some cases of dysmenorrhœa, he says, the patient refers her monthly pain especially to the situation of the ovary. She points to a spot about two inches internal to the anterior superior iliac spine, and says the pain is there; she may add that it goes through to the back, indicating a spot over the sacro-iliac synchondrosis of the same side, and when the fingers are pressed down upon the spot pointed to, the patient manifests more pain than when pressure is applied anywhere else. Sir James Simpson applied to cases presenting this kind of monthly pain the name of "ovarian dysmenorrhœa." This term, however, is nothing but a name—a label. It conveys no information and helps in no way in treatment. It would seem to imply that the pain is due to some condition of the ovary, and a hypothetical pathology of the ovary has been constructed to account for such cases. Various morbid conditions have been described which have been supposed to cause pain, such as "cirrhosis," "congestion," "œdema," "dropsy of follicles," etc. But in the best known and most conspicuous disease of the ovary—cystic disease—there is as a rule no pain until the cyst is big enough to press upon something else, and as to the other ill-marked morbid conditions which are supposed to cause such suffering, "all I need tell you," says Dr. Herman, "is that if you were to put upon a plate half a dozen ovaries, not much enlarged or adherent, removed from women who had this ovarian pain, and put with them half a dozen other ovaries taken from the bodies of women who were accustomed to menstruate without pain, there are no criteria by which anyone could pick out the ovaries of those who had pain when they menstruated. So long as we cannot distinguish a painful ovary from one which is not painful, it is idle to say that we know anything of the morbid conditions of the ovary upon which pain depends."

[1] Brit. Med. Jour., May 17.

